# Homogeneity of Arabian Peninsula dromedary camel populations with signals of geographic distinction based on whole genome sequence data

**DOI:** 10.1038/s41598-021-04087-w

**Published:** 2022-01-07

**Authors:** Hussain Bahbahani, Faisal Almathen

**Affiliations:** 1 Department of Biological Sciences, Faculty of Science, Kuwait University, Al-Shidadya, Kuwait; 2 Department of Public Health, College of Veterinary Medicine, King Faisal University, 400, Al-Hasa, Kingdom of Saudi Arabia; 3 Camel Research Center, King Faisal University, 400, Al-Hasa, 31982 Kingdom of Saudi Arabia

**Keywords:** Evolution, Genetics, Zoology

## Abstract

Dromedary camels in the Arabian Peninsula distribute along different geographical and ecological locations, e.g. desert, mountains and coasts. Here, we are aiming to explore the whole genome sequence data of ten dromedary populations from the Arabian Peninsula to assess their genetic structure, admixture levels, diversity and similarity indices. Upon including reference dromedary and Bactrian camel populations from Iran and Kazakhstan, we characterise inter-species and geographic genetic distinction between the dromedary and the Bactrian camels. Individual-based alpha genetic diversity profiles are found to be generally higher in Bactrian camels than dromedary populations, with the exception of five autosomes (NC_044525.1, NC_044534.1, NC_044540.1, NC_044542.1, NC_044544.1) at diversity orders (q ≥ 2). The Arabian Peninsula camels are generally homogenous, with a small degree of genetic distinction correlating with three geographic groups: North, Central and West; Southwest; and Southeast of the Arabian Peninsula. No significant variation in diversity or similarity indices are observed among the different Arabian Peninsula dromedary populations. This study contributes to our understanding of the genetic diversity of Arabian Peninsula dromedary camels. It will help conserve the genetic stock of this species and support the design of breeding programmes for genetic improvement of favorable traits.

## Introduction

The domestic one-humped *Camelus dromedarius* (dromedary camel) and two-humped *Camelus bactrianus* (Bactrian camel), in addition to the wild two-humped *camelus ferus*, form the old world Camelini tribe of the Camelidae family. The ancestors of this tribe, which diverged from the new world Lamini tribe about 16.3 million years ago (Mya), reached Eurasia via the Bering land bridge approximately 6.5–7.5 Mya. After the divergence of the one-humped camels from the two-humped animals, around 4.4 Mya, the Bactrian camels were domesticated from their wild ancestors about 5000–6000 years ago, most probably in eastern Asia. This was followed by the domestication of the dromedary camels in the southeastern region of the Arabian Peninsula around 3000–4000 years ago^1^.

Dromedary and Bactrian camels show wide geographical distribution due to their historical use in long-distance trading and transportation. Dromedary camels are predominantly found in the desert and semi-arid regions of Africa, Arabian Peninsula and southwest Asia, while Bactrian camels are mainly distributed throughout eastern and central Asia^2^. In several countries, including for instance Iran, India, Turkey and Kazakhstan, both domestic camel types can be found, and anthropogenic hybridization between them can result in animals with high robustness and endurance—commonly used along long-distance trade routes^1^.

In the Arabian Peninsula, dromedary camels are classified according to various criteria, for example coat color, ecological location and their productivity, however there is no established breeding system informed by genetic analysis. Regarding coat color, camels with dark brown to black coat colors are known as Magaheem, while camels with white coats are known as Wodeh. Sofor and Shual camels have a brown coat color, with Sofor camels mainly characterized as being dark brown to black at the top of their neck, shoulder, hump and tail^3^. All of the above mentioned camel populations are used for milk and meat production, are hence classified as production camels, and can be found in the north and central area of the Arabian Peninsula. Camels bred specifically for competitive racing are hence known as racing camels, and are mainly from either Oman in the southeast of the Arabian Peninsula, or the north of the Arabian Peninsula where a recognized population known as al-Hurra is popular^4,5^.

Dromedary camels from the west, e.g. Hadana and Sahlia, and southwest of Arabian Peninsula, e.g. Awadi and Awarik, face different ecological conditions than those from the north and center of the Arabian Peninsula. In contrast to the wide desert covering the north and center of the Arabian Peninsula, Hadana and Awadi camels are populating higher elevations, on the tops mountains, and are hence known as Hill or Mountain camels. By contrast, Sahlia and Awarik camels are mainly found near the Red Sea coast of Saudi Arabia and are known as Beach camels^5^.

Assessing the genetic diversity and structure of dromedary camels is the first milestone towards establishing a standard genetically informative breeding programme. Until recently this field of research has largely been confined to the analysis of autosomal microsatellite markers and partial mitochondrial DNA (mtDNA) sequences^6–8^, while more recent efforts by Bahbahani et al.^9^ and Ming et al.^10^ employed genotype-by-sequence (GBS) and whole genome sequence data, respectively. A fundamental challenge in assessing the genetic diversity of dromedary populations concerns accessibility to camel samples representing the different populations, and defining informative statistics with which to evaluate their diversity. Several diversity indices exist that measure species diversity at ecological levels, which can also be used to evaluate the genetic diversity of livestock species^11,12^. Shannon’s entropy, Simpson’s index and Renyi’s entropy are examples of such indices, which translated mathematically into number of equivalents called Hill numbers, behave in a way that can be used to express diversity^11^. All of these indices decompose into two components; alpha and beta diversities, which describe different diversity angles. Alpha diversity measures the average diversity of a single ecological community, while beta diversity measures the relative change in species composition between ecological communities^11^. An important parameter that differs between the Hill numbers of these two diversity components is the diversity order (q), which determines the sensitivity of the measure to the frequency of the species^13^. At q = 0, the species frequency, or abundance, is not counted. While, at q = 1, the species are weighed in proportion to their frequency. At q > 1, the Hill number becomes sensitive to the most frequent species^11,13^.

Recently, Ma et al.^12^ applied these ecological diversity measures to quantify SNP diversity at the level of the individual. These proposed alpha and beta diversity measures are able to account for the uneven, non-random, distribution of SNPs along chromosomes. Ma et al.^12^ proposed four Hill-number-based similarity measures to compare SNP similarity between populations. These measures investigate SNPs similarities at different levels: local SNP overlap (Cq_N_) that quantifies the proportion of shared SNPs among individuals; regional SNP overlap (UqN) that looks at the proportion of shared SNPs in the pooled population; SNP homogeneity (Sq_N_) that measures SNP evenness between populations; and SNP turnover complement (Vq_N_) that represents the relative rate of SNP turnover per individual.

In this study, we investigate if the different Arabian Peninsula dromedary camel populations can be genetically discriminated based on their geographical distribution using whole genome sequence data. Individual-level diversity and similarity indices are assessed in these populations and compared to reference dromedary and Bactrian camels.

## Results

### Summary statistics of mapped sequence reads

The mean depth of coverage of the mapped sequence reads among the dromedary samples ranged from 13.7 to 32.16 ×, with a mean of 25 ×, while among Bactrian camels it ranged from 5.96 to 23.02 × with a mean of 13.3 × (Supplementary Table S1). On average, 99.82% of the dromedary sequence reads and 99.61% of Bactrian sequence reads mapped to the dromedary reference genome (CamDro3), with 94.4% and 94.7% of mapped reads remaining properly paired in dromedaries and Bactrians, respectively. The average proportions of reference genome covered by the mapped reads were 93.25% in dromedaries and 90.84% in Bactrians (Supplementary Table S1).

### Genetic structure and relatedness

**Figure 1:**
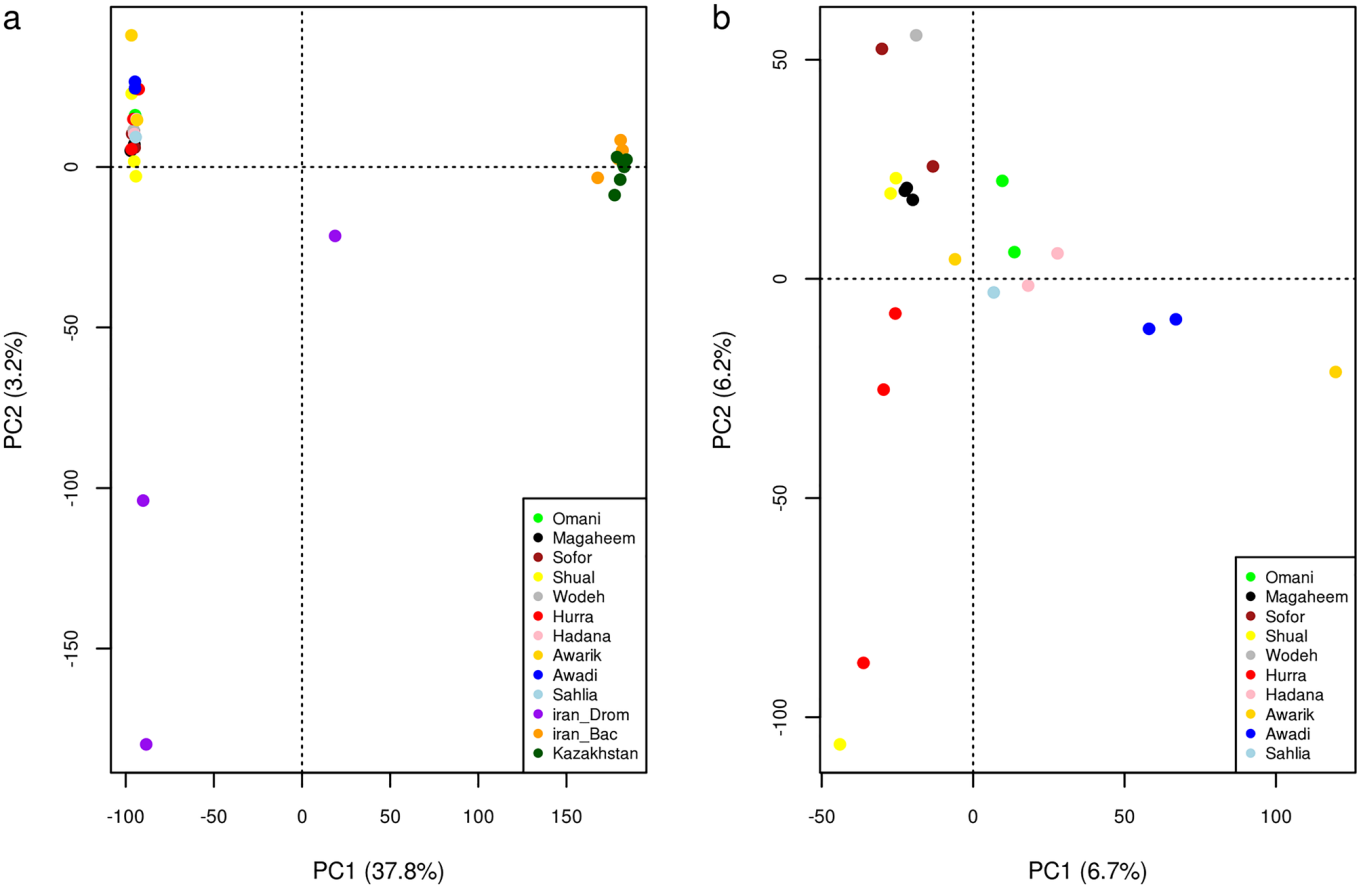
Principle component analysis (PCA) plots on (**a**) dromedary and Bactrian camels and (**b**) dromedary camels from the Arabian Peninsula.

Principle component analyses (PCA) analysis of all camels (dataset 1) reveals clear differentiation between dromedary and Bactrian populations along the first principle component (PC1), explaining 37.8% of the total variation. Whilst, the second principle component (PC2), accounting for 3.2% of the total variation, separates camels from Iran and the Arabian Peninsula populations (Fig. 1a).

A PCA focused only on the Arabian Peninsula dromedary camels (dataset 2) reveals structure broadly consistent with the geographic sampling. Dromedaries sampled from the west, southwest and southeast of the Peninsula (Hadana, Sahlia, Awarik, Awadi and Omani) are separated from those sampled from the north and center of the Arabian Peninsula populations (Hurra, Sofor, Shual, Wodeh, Magaheem) along PC1, explaining 6.7% of the total variation. PC2, which accounts for 6.2% of the total variation, shows some separation of the Hurra samples and Awadi camels from the other populations, in addition to an Awarik and a Shual samples (Fig. 1b).

The mean relatedness Ф between the Arabian Peninsula dromedary samples is 0.108 ± 0.094 indicating them to be second-degree relatives (Supplementary Table S2). A single first-degree relationship was identified between a Hurra and a Shual, which are separated from their corresponding populations as revealed by the PCA plot on Arabian Peninsula dromedary camels (Fig. 1b).

**Figure 2:**
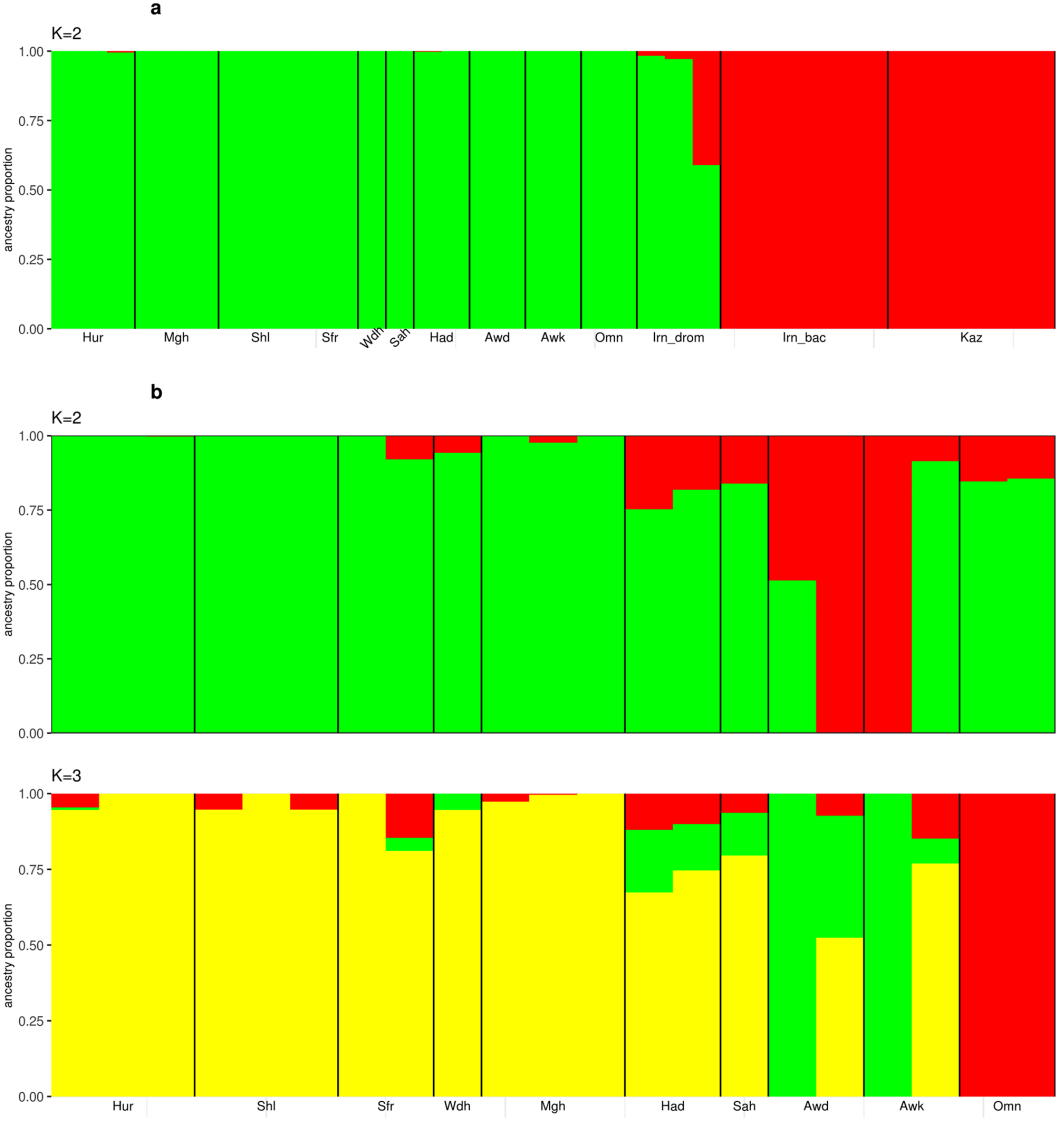
Admixture analysis plots of (**a**) *K* = 2 on the dromedary and Bactrian camels (dataset 1) and (**b**) *K* = 2 and 3 on the dromedary camels from the Arabian Peninsula (dataset 2). *Hur* Hurra, *Mgh* Magaheem, *Shl* Shual, *Sfr* Sofor, *Wdh* Wodeh, *Sah* Sahlia, *Had* Hadana, *Awd* Awadi, *Awk* Awarik, *Omn* Omani, *Irn_drom* Dromedary from Iran, *Irn_Bac* Bactrian from Iran, *Kaz* Bactrian from Kazakhastan.

Admixture analyses were performed to further investigate the genetic structure of both dromedary and Bactrian camels. The optimal number of clusters identified in dataset 1 (Fig. 2a) was *K* = 2 (Supplementary Table S3a). This *K* value differentiates between the dromedary and Bactrian camels. At this *K* value the dromedary camels from Iran share genetic background with Arabian Peninsula dromedaries, with one sample shows substantial Bactrian genetic introgression with ancestry proportion value ~ 0.43. A signal of distinct genetic background corresponding to dromedaries from Iran has emerged at *K* = 3, which is more clearly revealed at *K* = 5. At *K* = 4, a separate genetic ancestry is revealed for the Bactrian camels from Iran, with the exception of two samples carrying a substantial genetic background of Kazakhstan ancestry (ancestry proportion > 0.8) (Supplementary Fig. S1).

Admixture analysis of dataset 2 (Fig. 2b) reveals the optimal number of clusters to be *K* = 1 (Supplementary Table S3b). At *K* = 2, distinct genetic background emerges for dromedary camels from the southwest of the Arabian Peninsula; Awadi and Awarik camels, with the exception of a single Awarik camel mainly carrying a proportion of genetic ancestry (~ 0.92) not related to the southwest of Arabian Peninsula. A substantial proportion of the southwest genetic ancestry is observed in camel populations from the west; Hadana (~ 0.21) and Sahili (~ 0.16), and southeast; Omani (~ 0.15), of the Arabian Peninsula. At *K* = 3, a genetic background specific to Omani camels from the southeast of the Arabian Peninsula is revealed (Fig. 2b). Beyond *K* = 3 varying levels of admixture across most camels are observed (Supplementary Fig. S2).

### Individual-level SNP diversity

**Figure 3:**
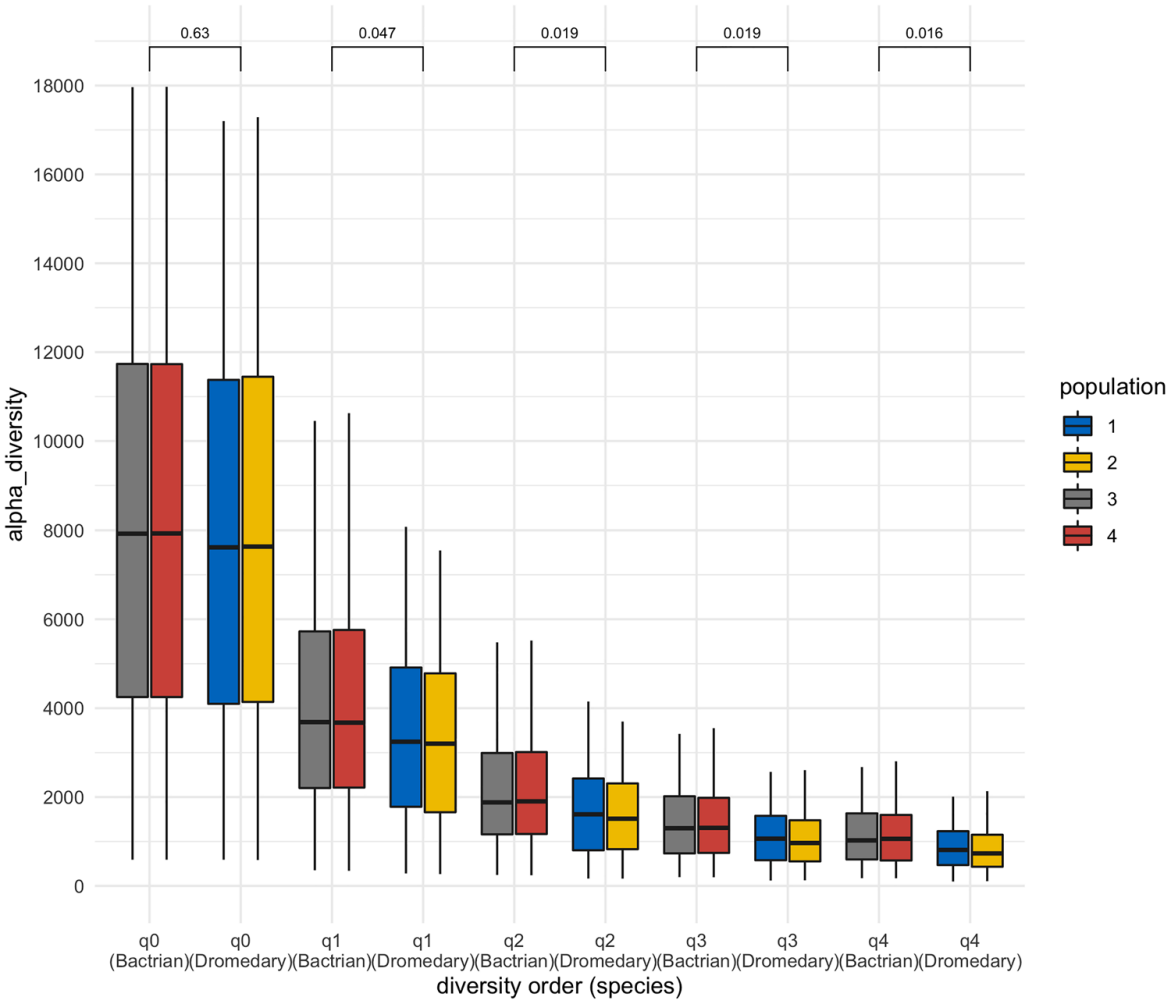
Mean autosomal alpha diversity at the diversity orders q = 0 to 4. Populations: (1) Arabian Peninsula dromedary camels; (2) dromedary camels from Iran; (3) Bactrian camels from Iran; and (4) Bactrian camels from Kazakhstan. Mann–Whitney *U* test *P*-values for pairwise comparisons of dromedary (1 and 2) against Bactrian (3 and 4) are reported.

Autosomal SNP diversity profiles were generated for each camel to enable alpha diversity to be calculated at the individual-level. Alpha diversity was observed to be significantly higher (Mann–Whitney *U* test *P*-value < 0.05) in Bactrian camel populations than in dromedary camels at all diversity orders, except q = 0 (Fig. 3; Supplementary Table S4). No statistically significant differences in the average autosomal alpha diversity are observed between dromedary populations, or between Bactrian Populations (Supplementary Table S4). On average, we observe higher alpha diversities in Bactrian camel populations on all but five autosomes, with these exceptions showing significantly higher diversity values in dromedaries at diversity orders q ≥ 2 (Table 1; Supplementary Table S5; Supplementary Fig. S3). Among the Arabian Peninsula dromedaries, no significant difference in alpha diversity is observed among the different populations for any of the diversity orders tested (Supplementary Fig. S4; Supplementary Table S6).

**Table 1:** Mann–Whitney *U* test *P*-values for autosomes showing significantly higher alpha diversities in dromedaries than Bactrian camels at different diversity orders (q).

Autosome	q0_P_value	q1_P_value	q2_P_value	q3_P_value	q4_P_value
NC_044525.1	1.0000	0.9996	0.0285	0.0003	0.0000
NC_044534.1	1.0000	0.1200	0.0000	0.0000	0.0000
NC_044540.1	1.0000	1.0000	1.0000	0.9776	0.0025
NC_044542.1	1.0000	1.0000	0.2661	0.0006	0.0001
NC_044544.1	1.0000	0.9998	0.1888	0.0191	0.0148

**Figure 4:**
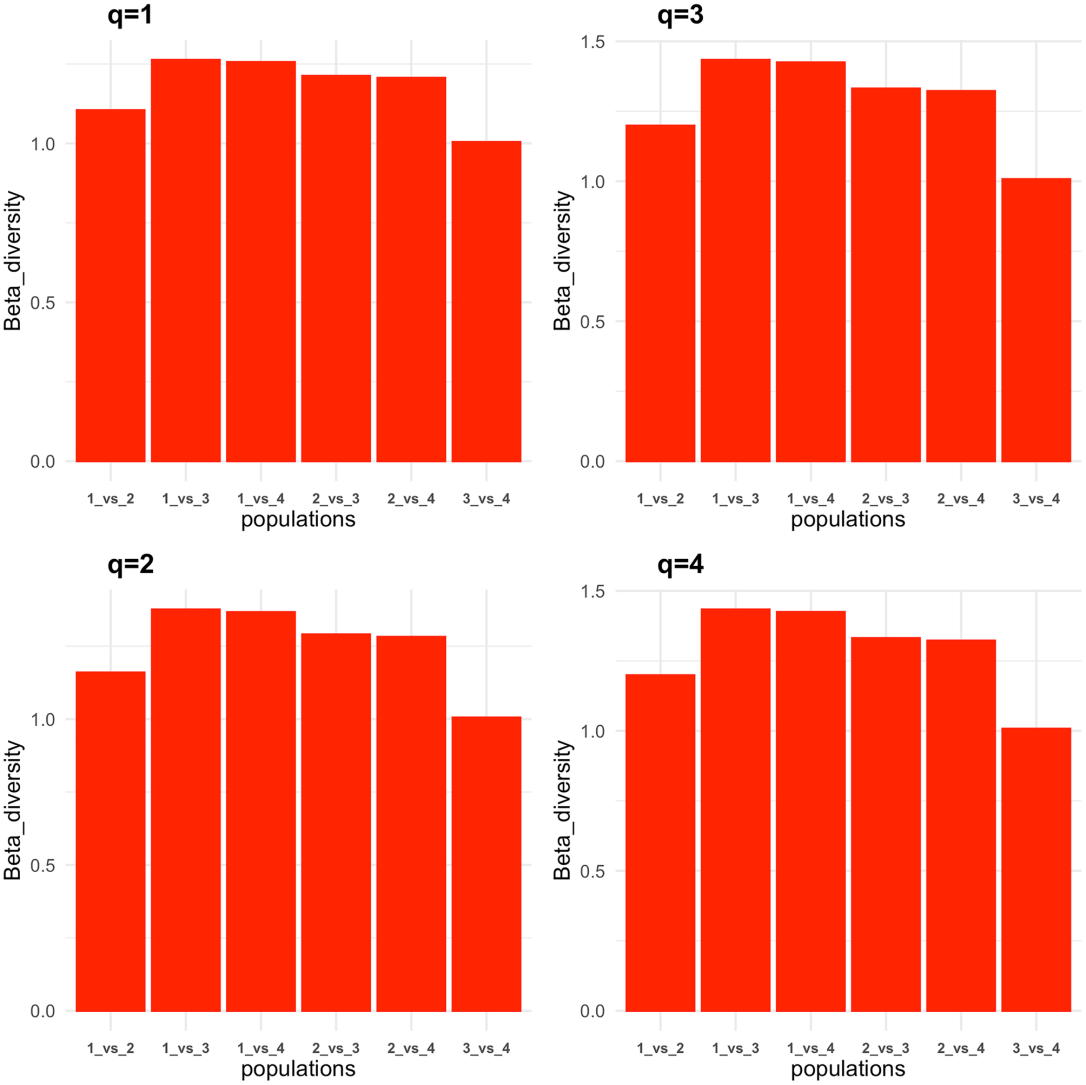
Average autosomal beta diversity at diversity orders q = 1 to 4. Populations: (1) Arabian Peninsula dromedary camels; (2) dromedary camels from Iran; (3) Bactrian camels from Iran; and (4) Bactrian camels from Kazakhstan.

**Figure 5:**
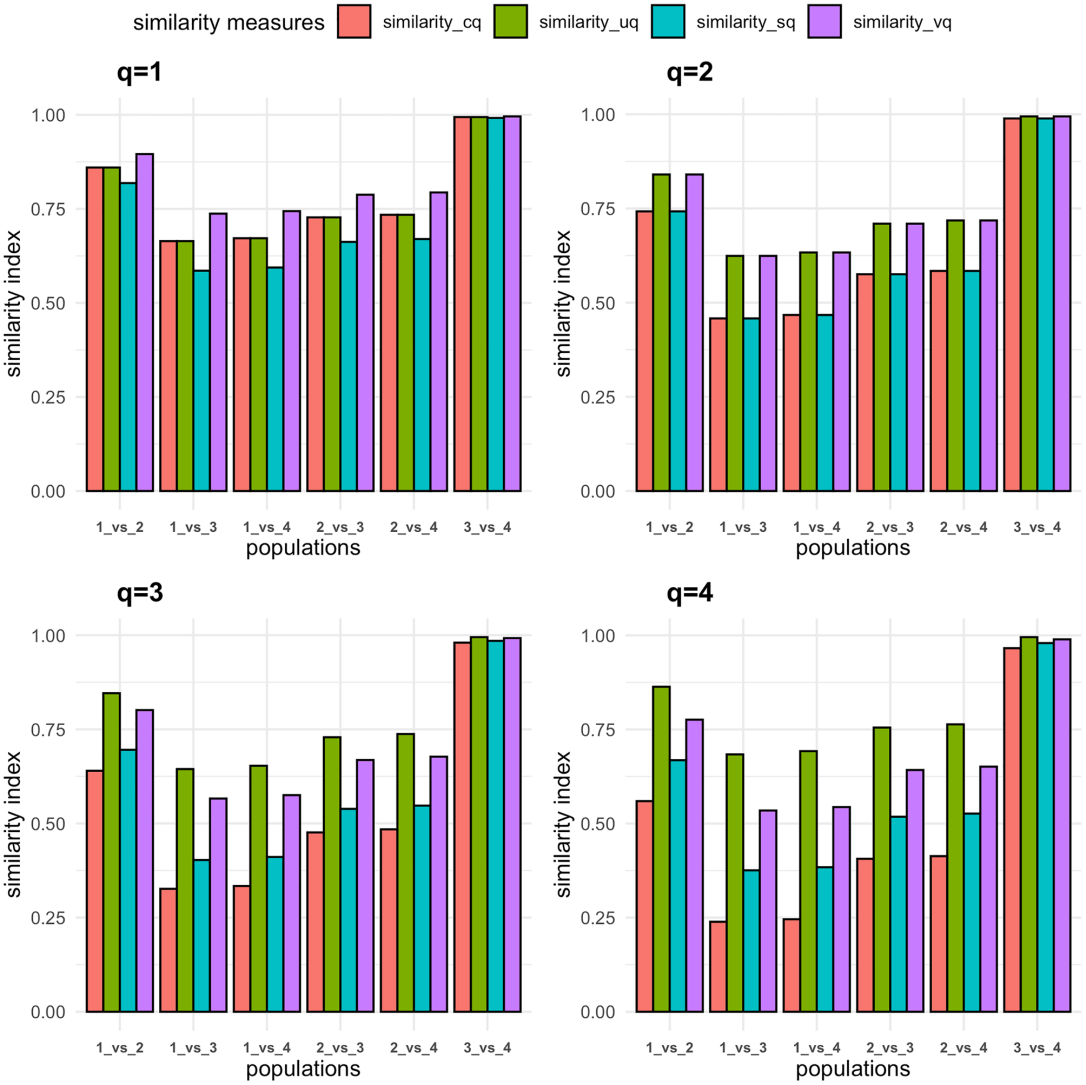
Average autosomal similarity indices at diversity orders q = 1–4. Populations: (1) Arabian Peninsula dromedary camels; (2) dromedary camels from Iran; (3) Bactrian camels from Iran; and (4) Bactrian camels from Kazakhstan.

To further characterise population differentiation, beta diversity and similarity indices were calculated within and between populations for diversity orders q = 0 to 4. Analysing dataset 1, we compared: (1) dromedary from the Arabian Peninsula; (2) dromedary from Iran; (3) Bactrian camels from Iran; and (4) Bactrian camels from Khazakhstan. At diversity orders q ≥ 1, We observed higher beta diversity and lower similarity indices when comparing between species (mean beta = 1.34, Cq = 0.49, Uq = 0.70, Sq = 0.52, Vq = 0.66), than within species (mean beta = 1.09, Cq = 0.84, Uq = 0.92, Sq = 0.86, Vq = 0.91) (Figs. 4 and 5; Supplementary Table S7). A comparison of dromedary from Iran to the Bactrian camels returned lower beta diversity and higher similarity indices (mean beta = 1.29, Cq = 0.55, Uq = 0.73, Sq = 0.58, Vq = 0.71) than when comparing dromedary from the Arabian Peninsula to Bactrian camels (mean beta = 1.38, Cq = 0.43, Uq = 0.65, Sq = 0.46, Vq = 0.62) at diversity orders q ≥ 1 (Figs. 4 and 5; Supplementary Table S7). Analysis of only the Arabian Peninsula dromedary camels (dataset 2) reveals no significant variation in beta diversity and similarity indices between the different populations at all diversity orders tested (Supplementary Table S8).

## Discussion

The inter-species genetic distinction observed between the dromedary and Bactrian camels revealed by both the PCA and admixture analysis likely results from more than 4 M years of divergence between these two species^1^. Evidence of hybridization between these species can be observed in countries where they coexist, such as Iran, Kazakhstan and Turkey. This genetic hybridization, which explains the introgressed Bactrian genetic ancestry into a dromedary sample from Iran at *K* = 2 of the dataset 1 admixture analysis, has previously been detected by Ming et al.^10^, and it is associated with breeding camels that combine the robustness and endurance of both species to withstand long-distance journeys^1^. The *nar* (male) and *nar-maya* (female) camels in Kazakhstan, and *tulu* camel in Turkey are examples of such cross-breeding as reviewed by Dioli^14^.

This level of inter-species genetic distinction is also evidenced in the individual-level alpha and beta diversity profiles and similarity indices. However, as the SNPs used in these analyses were derived from aligning sequencing reads to the dromedary camel reference genome assembly (CamDro3), it is inevitable that a greater number of SNPs would be identified in the more divergent species, resulting in higher alpha diversity values in Bactrian camels. Nonetheless, we observed higher alpha diversity profiles in dromedary camels than Bactrians on five autosomes, which may indicate that diversity in the genes on these autosomes may be of functional significance to dromedary camels. This is an interesting observation that warrants further investigation, for example using signatures of selection and gene-wise association analyses with potentially relevant traits.

We observed a degree of differentiation by PCA, admixture and diversity analyses, between the dromedaries from the Arabian Peninsula and those from Iran, which may further be clarified upon including more dromedary samples from Iran. This differentiation is likely resulted from geographical separation by the sea preventing gene flow. Such geographical-wise genetic distinction, has also been observed among African dromedary populations, from Algeria and Egypt^15^, and between African and Arabian Peninsula dromedaries^9^.

The Arabian Peninsula dromedaries appear to be a homogenous population based on PCA, admixture and diversity analyses. Such genetic homogeneity is likely a consequence of high genetic admixture between the subpopulations sampled, possibly driven by the historical use of dromedaries in trading and transportation along the Arabian Peninsula^16^. Another possible factor contributing to gene flow are the breeding practices of camel owners in the Arabian Peninsula, which are generally panmictic, lacking a structured breeding programme such as that typically observed in other livestock^17^. The level of admixture between Arabian Peninsula dromedaries obtained here can explain the observed high relatedness between the dromedary samples in the area. Despite this homogeneity, admixture analyses at *K* = 2 and *K* = 3 suggest a degree of genetic structure associated with geography, with dromedaries falling into three groups: (1) North (Hurra, Shual, Wodeh and Sofor), Central (Magaheem), West (Hadana and Sahilia); (2) Southwest (Awarik and Awadi); and (3) Southeast (Omani). Such level of genetic structuring has previously been observed on Arabian Peninsula camels using microsatellite genotyping^6,7,18^.

The north and center of the Arabian Peninsula is mainly characterized by plain desert, while the west and southwest of the Arabian Peninsula is characterized by variability in elevation associated with mountains, reaching 3000 m in altitude^19^, and the coast of the Red Sea. This variable ecology might contribute to genetic differentiation between the dromedary camels populating the different areas. Although the west and southwest camel populations show a degree of genetic differentiation, their close proximity and likelihood of continuous gene flow may explain the shared genetic ancestry proportion between them. For example, the Awadi camels, which are typically found in the mountains of the Jazan region in the southwest, may also be found along the Red sea coast where Sahlia and Awarik camels are distributed.

The calculated diversity profiles in this study are associated with different advantages benefiting livestock population genetics studies. First, unlike the well-known population-level diversity measures, e.g. heterozygosity, that require large number of samples per population, they are calculated at individual level solving the problem of low number of samples available per population. Second, both of the alpha and beta profiles assess the distribution pattern of the SNPs along each chromosome, or the whole genome, which other diversity measures do not. Third, these profiles can be calculated using other genetic variations, such as insertions, deletions and inversions^12^.

## Conclusion

We have presented here the first whole-genome sequence analysis of the genetic structure and diversity of Arabian Peninsula dromedary camels. By including dromedary and Bactrian reference populations from outside of the Arabian Peninsula, inter-species and geographic genetic differentiations have been revealed. The Arabian Peninsula camel appear to be a homogenous gene pool with a subtle degree of geographic structure. To validate these findings a larger cohort of camels from populations spanning the whole of the Peninsula is necessary. This study is a first step towards understanding the genetic diversity of dromedaries in the Arabian Peninsula, which is known as their center of domestication.

## Materials and methods

### Dromedary samples collection and whole genome DNA extraction

A total of 5 ml blood sampled from the jugular vein was collected from each of 21 dromedary camels using standard techniques approved and in accordance with the relevant guidelines and regulations of the department of veterinary public health and animal husbandry at King Faisal university (Ref: KFU-REC/2018-10-01). All authors compiled with the ARRIVE guidelines 2.0^20^. These samples represent dromedary populations from several geographical locations in the Arabian Peninsula (Table 2; Supplementary Table S9). Genomic DNA was extracted using the DNeasy^®^ Blood and Tissue kit (Qiagen) according to the manufacturer’s protocol.

**Table 2:** Dromedary camel populations from Arabian Peninsula included in the study, their geographical distribution and the number of samples in each population.

Population	Geographical location in the Arabian Peninsula	Number of samples
Hurra	North	3
Shual	North	3
Sofor	North	2
Wodeh	North	1
Magaheem	Center	3
Hadana	West	2
Sahlia	West	1
Awadi	Southwest	2
Awarik	Southwest	2
Omani	Southeast	2

### Whole genome sequence data processing

Genomic DNA was sequenced using paired-end libraries on an Illumina Hiseq 2000 platform at Macrogen in South Korea. Publicly available whole-genome sequence data for four dromedary camels from Iran, six Bactrian camels from Iran, and six Bactrian camels from Kazakhstan (Supplementary Table S9) generated by Ming et al.^10^ was downloaded from NCBI’s Sequence Read Archive (https://www.ncbi.nlm.nih.gov/sra).

The fastp software version 0.22.0^21^ was used to trim adapters from the raw sequence reads of the Arabian Peninsula dromedary samples. Raw sequence reads were discarded if: (1) 10% or more of the read bases were uncertain; (2) 40% or more of the read bases were low quality (base Q_phred_ ≤ 20); (3) reads length was shorter than 15 bases; or (4) read complexity was less than 30%. Bases with quality score (Q_phred_) less than 20 were also filtered out from the reads. The remaining high-quality reads were mapped against the African dromedary reference genome assembly (CamDro3)^22^ using the BWA-MEM algorithm implemented in Burrows–Wheeler Aligner (BWA)^23^. Reads were coordinate-sorted using the *SortSam* option, and PCR-duplicates were marked and excluded using the *MarkDuplicates* and *REMOVE_DUPLICATES* = *true* options in the Picard tools version 1.119 (http://broadinstitue.github.io/picard/index.html). Summary statistics calculated for mapped reads included: the proportion of reference genome coverage via the *genomeCoverageBed* option implemented in BEDTools software version 2.17^24^; mean depth of coverage via the DepthOfCoverage tool implemented in GATK version 3.6^25^; and the total number and percentage of mapped reads via the flagstat tool in SAMTools software version 1.19^26^.

### Variant calling and filtering

Single Nucleotide Polymorphisms (SNPs) were called from the mapped sequence reads using the GATK version 4.1.4.0 *HaplotypeCaller* tool in *GVCF* mode. Variants were subsequently combined and genotyped using the GATK *CombineGVCFs* and *GenotypeGVCFs* tools in two datasets: dataset 1, which comprised all camel samples; and dataset 2, which comprised only the dromedary camels from the Arabian Peninsula. Quality control filtering criteria were applied on the SNPs of each dataset using the GATK *VariantFilteration* tool. Parameters included: (1) excluding variants with low quality by depth (QD) (QD < 2); (2) excluding variants with root mean square of mapping quality for all reads of a site less than 40 (MQ < 40.0); (3) excluding variants with base quality score less than 30 (QUAL < 30); (4) excluding variants with high probability of allele-specific strand bias between forward and reverse strand (FS > 60); (5) excluding variants with bias in mapping quality between the reads supporting the reference and alternative alleles (MQRankSum < − 12.5); and (6) excluding variants with bias in the position of the alternative allele towards the ends of the reads (ReadPosRankSum < − 8). SNPs with a depth of coverage ranging between two reads and three standard deviations from the mean depth of coverage across samples were retained. A total of 14,224,566 and 4,945,503 autosomal SNPs were retained for dataset 1 and dataset 2, respectively, which are used to calculate SNP diversity and similarity indices.

For the admixture and principal components analyses (PCA), SNPs were further filtered with Plink 1.9^27^. Within each dataset, SNPs were excluded if: (1) they had a minor allele frequency ≤ 5%; (2) had a call rate ≤ 95%; or (3) departed significantly from Hardy–Weinberg equilibrium (*p*-value < 1 × 10^–6^). SNPs were also filtered to exclude those with a linkage disequilibrium (LD) correlation coefficient r^2^ value > 0.1, as in Bahbahani et al. (2019), using Plink’s *indep-pairwise* tool (--indep-pairwise 50 kb 10 kb 0.1) resulting in 44,962 SNPs in dataset 1 and 58,617 SNPs in dataset 2 (Table 3).

**Table 3:** Number of autosomal SNPs excluded by filters in dataset 1 (all camel populations) and dataset 2 (Arabian Peninsula dromedary populations).

	Datasets	
	Dataset 1	Dataset 2
Raw SNPs number	14,224,566	4,945,503
**Quality control criteria**		
MAF < 5%	2,396,749	1,237,037
Call rate < 95%	1,951,013	63,562
HWE (p-value < 1 × 10^–6^)	3,357,791	24
LD (r^2^ > 0.1)	6,474,051	3,586,263
Final number of SNPs retained	44,962	58,617

Samples were excluded if: (1) their genotyping call rate was ≤ 95%; or (2) they shared identity-by-state (IBS) ≥ 95% with another sample, in which case that the lowest genotyping call rate was excluded. A single dromedary camel sample from Iran (SRR5563498) was excluded from dataset 1 due to high IBS with another dromedary sample from Iran (SRR5563500).

### Genetic structure and relatedness

PCA was conducted using the *prcomp* function of R software^28^ on datasets 1 and 2 to determine the genetic relationship between the dromedary and Bactrian samples, and among the dromedary samples from the Arabian Peninsula. Local ancestry admixture analyses were conducted on both datasets using ADMIXTURE 1.23^29^, assuming the number of ancestral clusters (*K*) ranged from 1 to 13 for dataset 1, and *K* from 1 to 10 for dataset 2, which reflects the total number of sampled populations in each dataset. A total of 200 bootstrap iterations were performed for each *K* analysis. The optimal number of clusters was determined as the *K* value with the lowest cross-validation (CV) error.

Pairwise relatedness tests were conducted on dataset 2 using the *--relatedness2* tool implemented in VCFtools version 1.13^30^. This tool implements the Kinship-based INference for Genome-wide association studies (KING) algorithm^31^ to determine relatedness (Ф). A Ф > 0.354 indicates a duplicate sample or monozygotic twin, 0.177 < Ф < 0.354 indicates first-degree relatives, 0.0884 < Ф < 0.177 indicates second-degree relatives, 0.0442 < Ф < 0.0884 indicates third-degree relatives, while Ф < 0.0442 indicates the sample-pair to be unrelated^31^.

### Individual-level SNP diversity and similarity profiles

The diversity and similarity profiles, i.e. alpha and beta diversities and similarity indices, of autosomal SNPs per gene were calculated for each autosome in datasets 1 and 2 as detailed by Ming et al.^10^. For dataset 1, four populations were defined: (1) dromedary from Arabian Peninsula; (2) dromedary from Iran; (3) Bactrian from Iran; and (4) Bactrian from Kazakhstan. For dataset 2, the ten Arabian Peninsula dromedary populations were treated as separate populations since the admixture analysis did not reveal any clear clustering. Individual-level alpha diversity profiles were calculated at diversity orders *q* = 0 to 4. The mean alpha diversities were compared between populations using the non-parametric Mann–Whitney *U* test. For each diversity order, for all pairwise population comparisons in each dataset, we calculated beta diversity in addition to four similarity indices to investigate SNP variation levels: local SNP overlap (Cq); regional SNP overlap (Uq); SNP homogeneity measures (Sq); and SNP turnover complement (Vq).

## Supplementary Information

Supplementary Information.

Supplementary Table S1.

Supplementary Table S2.

Supplementary Table S3.

Supplementary Table S4.

Supplementary Table S5.

Supplementary Table S6.

Supplementary Table S7.

Supplementary Table S8.

Supplementary Table S9.

## Data Availability

The Arabian Peninsula dromedary whole genome sequence data analysed during the current study are available in the European Nucleotide Archive (ENA) with the Bioproject accession number (PRJEB47650).
