# Supplementary material for: Homogeneity of Arabian Peninsula dromedary camel populations with signals of geographic distinction based on whole genome sequence data
Source: Sci Rep. 2022 Jan 7;12:130. doi: 10.1038/s41598-021-04087-w (PMC8741945; doi:10.1038/s41598-021-04087-w)
Supplement: Supplementary file 1 — Supplementary Information. [file 41598_2021_4087_MOESM1_ESM.pdf]

# Homogeneity of Arabian Peninsula dromedary camel populations with signals of geographical distinction based on whole genome sequence data

**Supplementary material**

**Hussain Bahbahani[(1)*] and Faisal Almathen[(2,3)]**

[1]Department of Biological Sciences, Faculty of Science, Kuwait University, Kuwait.

[2]Department of Veterinary Public Health and Animal Husbandry, College of Veterinary Medicine, King Faisal University, 400 Al-Hasa, Kingdom of Saudi Arabia.

[3] Camel Research Center, King Faisal University, 400 Al-Hasa, 31982, Saudi Arabia.

*Corresponding author: hussain.bahbahani@ku.edu.kw

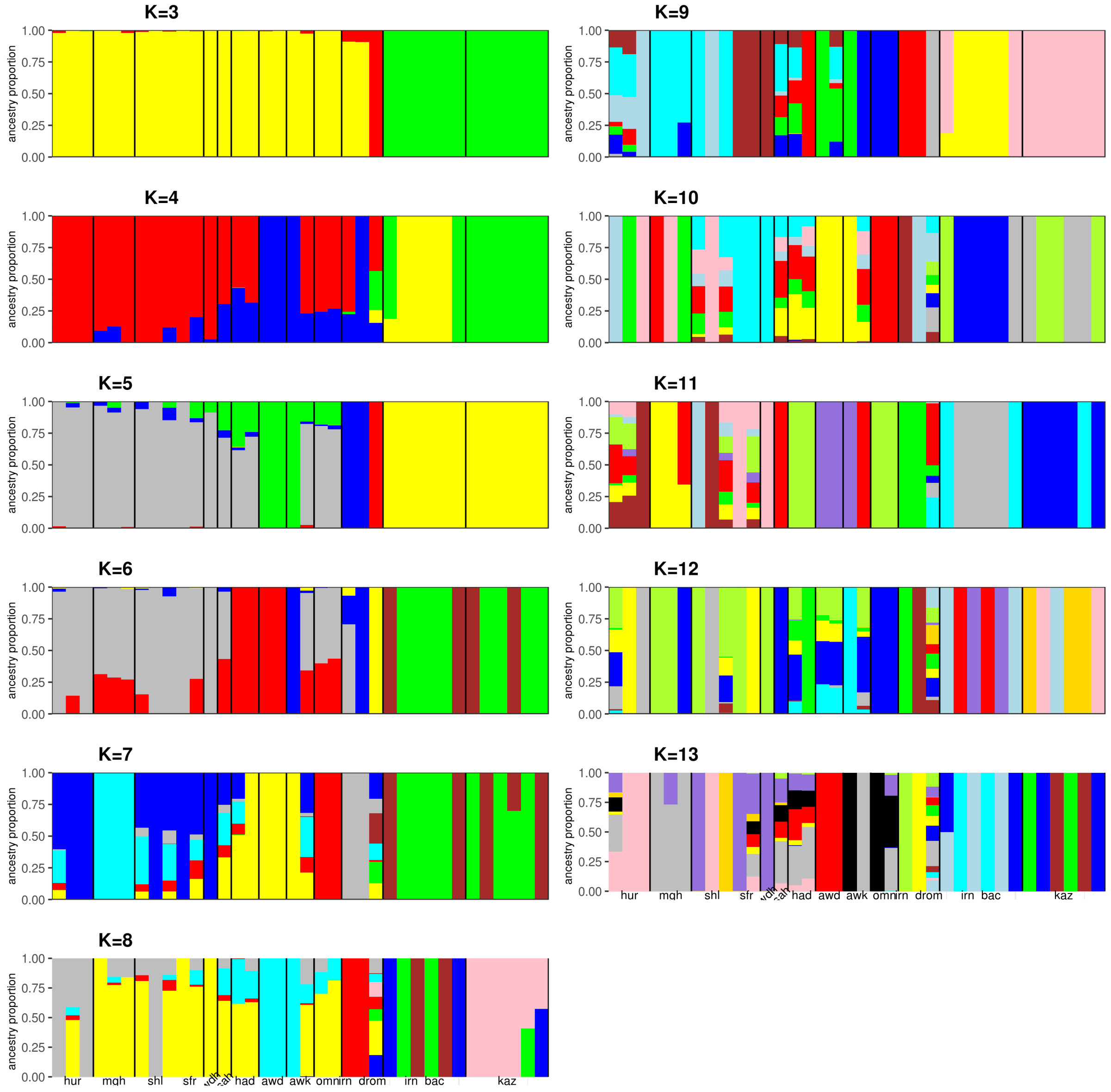


Supplementary Figure S1: Admixture analysis plots of $K$ = 3 to $K$ = 13 on the dromedary and Bactrian camels (dataset 1). hur: Hurra, mgh: Magaheem, shl: Shual, sfr: Sofor, wdh: Wodeh, Sah: Sahlia, had: Hadana, awd: Awadi, awk: Awarik, omn: Omani, irn_drom= Dromedary from Iran, irn_Bac = Bactrian from Iran, kaz = Bactrian from Kazakhastan.

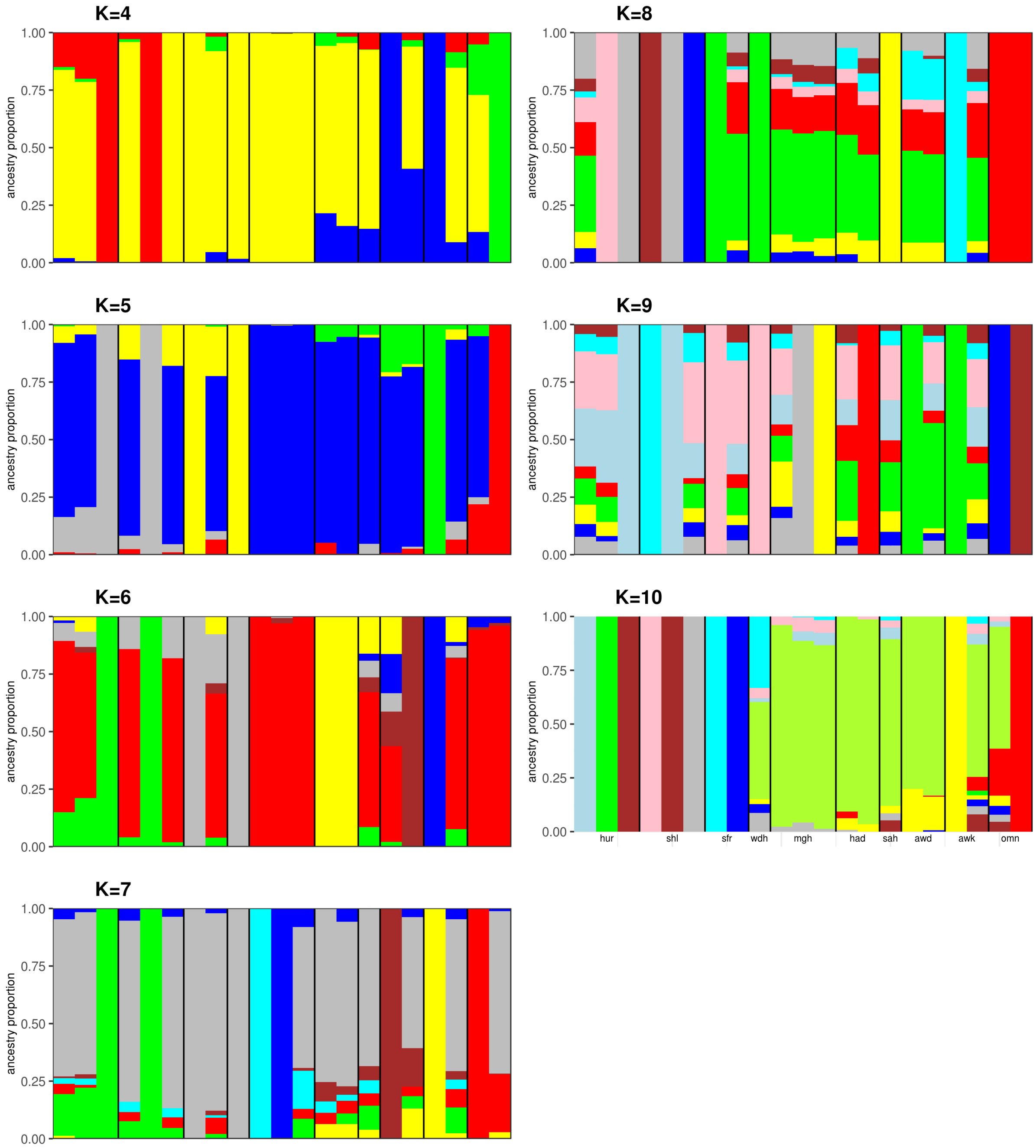


Supplementary Figure S2: Admixture analysis plots of $K$ = 4 to $K$ = 10 on the dromedary camels from the Arabian Peninsula (dataset 2). hur: Hurra, mgh: Magaheem, shl: Shual, sfr: Sofor, wdh: Wodeh, Sah: Sahlia, had: Hadana, awd: Awadi, awk: Awarik, omn: Omani.

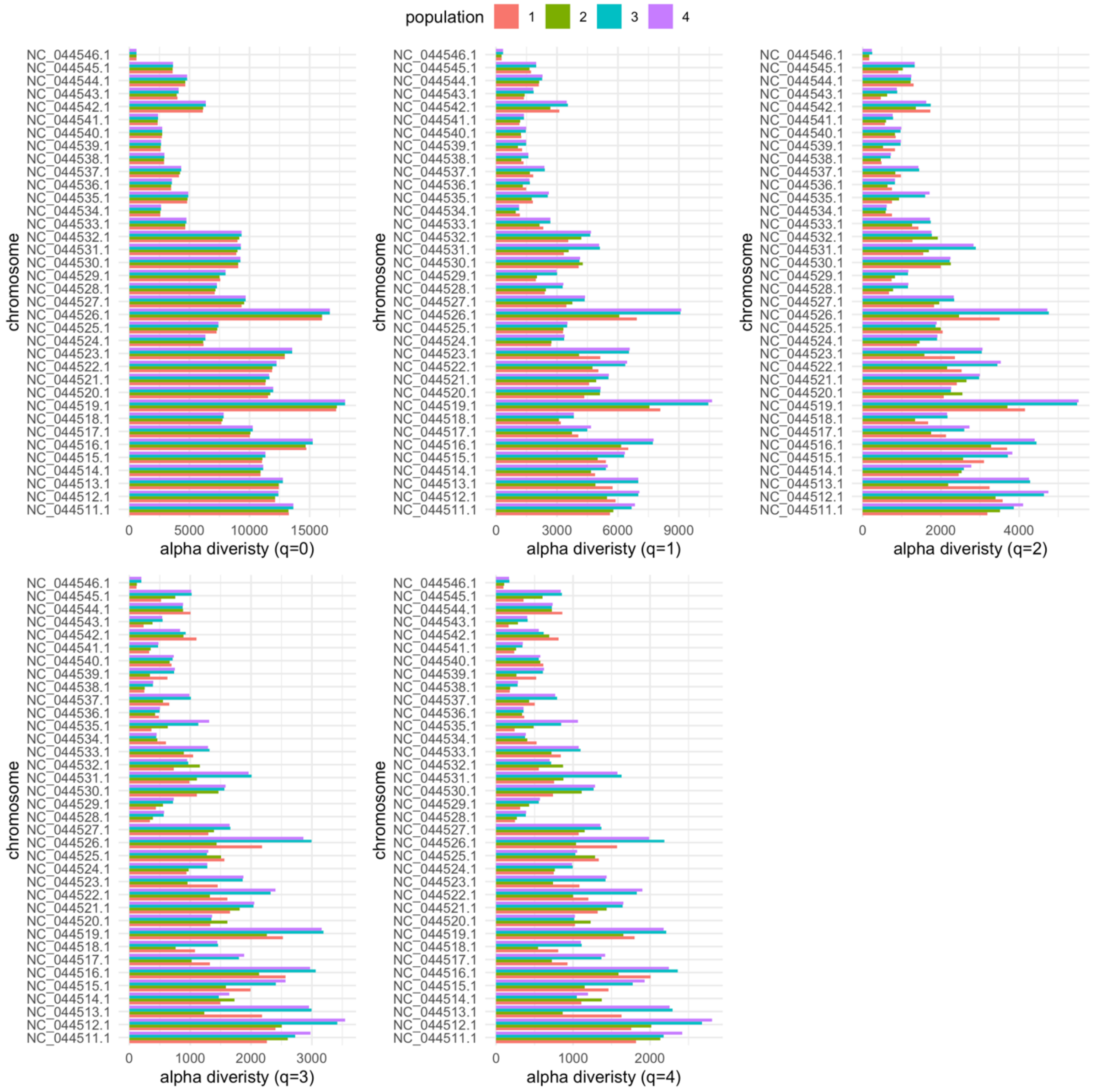


Supplementary Figure S3: Autosomal-wise alpha diversity at the diversity orders q = 0 to 4 on dromedary and Bactrian camels. Populations: (1) Arabian Peninsula dromedary camels; (2) dromedary camels from Iran; (3) Bactrian camels from Iran; and (4) Bactrian camels from Kazakhstan.

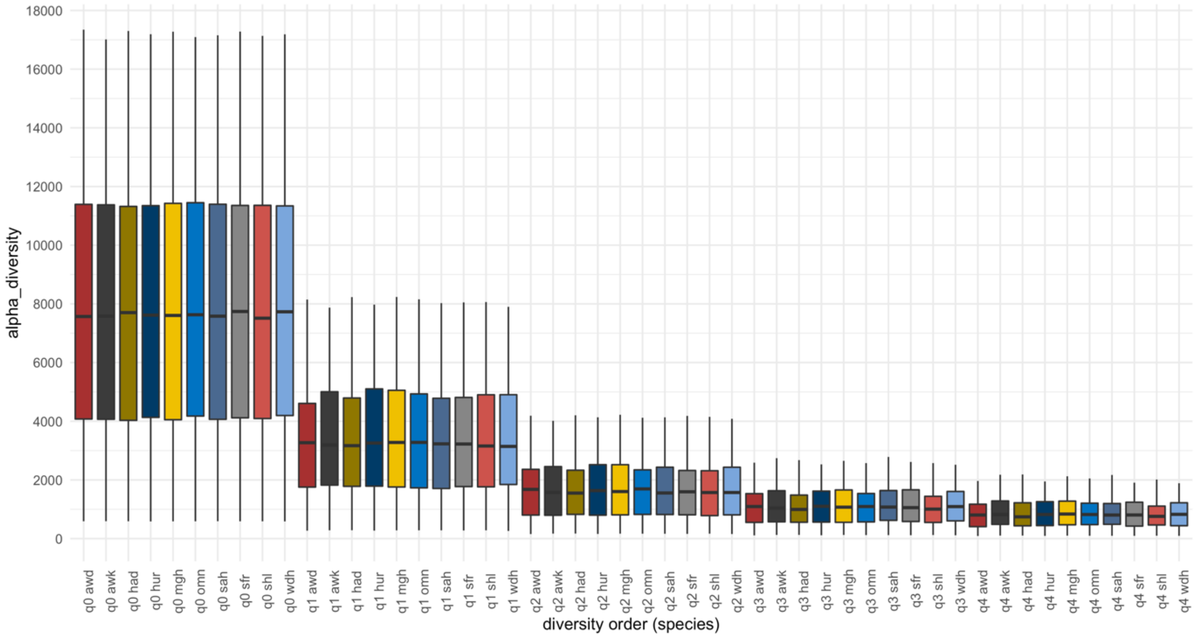


Supplementary Figure S4: Mean autosomal alpha diversity at the diversity orders q = 0 to 4 on Arabian Peninsula dromedary camel populations. hur: Hurra, mgh: Magaheem, shl: Shual, sfr: Sofor, wdh: Wodeh, Sah: Sahlia, had: Hadana, awd: Awadi, awk: Awarik, omn: Omani.

TableS1: summary statistics of the dromedary and Bactrain mapped sequence reads against CamDro3 reference genome assembly.

An excel file is submitted separately

Table S2: The relatedness value (Φ) of the different Arabian Peninsula dromedary pairs

An excel file is submitted separately

TableS3: cross-validation errors of the admixture runs for datasets 1 (A) and 2 (B). Highlighted $K$ values are the optimal values

An excel file is submitted separately

TableS4: Mann-Whitney U test P-value of the alpha diversity profile between the different populations in dataset 1.

An excel file is submitted separately

Table S5: Mann-Whitney U test P-values for all autosomes upon comparing alpha diversity between dromedary and Bactrian camel populations

An excel file is submitted separately

Table S6: Mann-Whitney U test P-values for the different Arabian Peninsula dromedary populations pairwise comparisons. Population labels: 1 = Omani, 2= Magaheem, 3= Sofor, 4= Shaul, 5= Wodeh, 6= Hurra, 8= Hadana, 9= Awarik, 10= Awadi, 11=Sahili

An excel file is submitted separately

TablsS7: The beta diversity and similarity indices at diversity orders (q = 0 - 4) for the different populations comparisons in dataset 1. Pop_1: Arabian Peninsula dromedaries. Pop_2: dromedaries from Iran, pop_3: Bactrian from Iran, pop_4: Bactrian from Kazakhstan

An excel file is submitted separately

Table S8: Beta diversity and similarity indices at diversity orders q = 0 to 4 for the Arabian Peninsula dromedary populations pairwise comparisons. Population labels: 1 = Omani, 2= Magaheem, 3= Sofor, 4= Shaul, 5= Wodeh, 6= Hurra, 8= Hadana, 9= Awarik, 10= Awadi, 11=Sahili

An excel file is submitted separately

Table S9: The sample ID (accession number of downloaded samples), origin and source of the dromedary and Bactrian camels included in the study

An excel file is submitted separately
